# Coding of Visual Object Features and Feature Conjunctions in the Human Brain

**DOI:** 10.1371/journal.pone.0003781

**Published:** 2008-11-21

**Authors:** Jasna Martinovic, Thomas Gruber, Matthias M. Müller

**Affiliations:** 1 School of Psychology, University of Liverpool, Liverpool, United Kingdom; 2 Institut für Psychologie, University of Osnabrück, Osnabrück, Germany; 3 Institut für Psychologie I, Universität Leipzig, Leipzig, Germany; Victoria University of Wellington, New Zealand

## Abstract

Object recognition is achieved through neural mechanisms reliant on the activity of distributed coordinated neural assemblies. In the initial steps of this process, an object's features are thought to be coded very rapidly in distinct neural assemblies. These features play different functional roles in the recognition process - while colour facilitates recognition, additional contours and edges delay it. Here, we selectively varied the amount and role of object features in an entry-level categorization paradigm and related them to the electrical activity of the human brain. We found that early synchronizations (approx. 100 ms) increased quantitatively when more image features had to be coded, without reflecting their qualitative contribution to the recognition process. Later activity (approx. 200–400 ms) was modulated by the representational role of object features. These findings demonstrate that although early synchronizations may be sufficient for relatively crude discrimination of objects in visual scenes, they cannot support entry-level categorization. This was subserved by later processes of object model selection, which utilized the representational value of object features such as colour or edges to select the appropriate model and achieve identification.

## Introduction

In the initial steps of the recognition process, an object's features are thought to be coded very rapidly in distinct but coordinated neural assemblies. The significance of the detail in the depiction of these features depends on the level of specificity at which object representation occurs: while superordinate classification (e.g., identifying an image of a bird as ‘an animal’) can be performed based on very few image qualities that roughly define shape, basic level (e.g., identifying it as ‘a bird’) or subordinate level (e.g., identifying it as ‘a penguin’) classification will rely on other features too. In everyday life, objects are recognised at the entry-level of recognition, which combines basic and subordinate levels. This means that while more typical exemplars will be recognised at basic-level (e.g., sparrow classed as a ‘bird’), exemplars with distinct attributes will be recognised at a subordinate level (e.g., ‘an ostrich’ or ‘a peacock’). Surface detail (texture, shading and colour) and visual complexity (intricacy of lines and detail) represent the two most obvious forms of object-image properties that can impact on entry-level recognition processes. Their functional roles differ - while colour facilitates recognition [1], additional amount of contours and edges delays it [2].

Theories of object recognition differ in the significance they attribute to various kinds of object's features. Some consider representations to rely mainly on shape [3], [4], while others also acknowledge the contribution of surface detail, such as colour [5]. Recent evidence supports the latter ‘shape and surface’ models that posit a genuine role of surface detail: colour, in particular, showing an advantage in recognition of objects [1], faces [6] and natural scenes [7]. Facilitative effects of colour on behavioural performance were recently confirmed in children [8] and illiterate adults [9], although it is often necessary to use presentation at threshold levels (i.e., masked brief images) in order to obtain behavioural effects of colour in normal adults. Colour is considered to be an aid in picture decoding or discrimination through improving access to stored object knowledge. Similarly, visual complexity is assumed to determine the ease of picture decoding, or the ease of processing before or at the structural stage of object recognition. However, opposite to colour, it has a detrimental effect on recognition performance [2].

Object features such as shape, colour or texture are coded very rapidly. Therefore, many researchers agree that an early stream of feature-coding neural processes must drive the speed of object recognition. Indeed, in cases when observers are looking for the presence of a particular object in natural scenes recognition can be ultra-rapid. For example, Thorpe, Fize and Marlot [10] have shown that approximately 150 ms is needed to identify the presence of an animal in a natural scene. The initial feedforward stream of up to 100–150 ms is thus sufficient for coarse object categorisation [11], [12]. It has been argued that this is due to an early activation of high-level units in the ventral visual stream – these units are assumed to select high-level feature conjunctions diagnostic for target-category stimuli [13]. In the aforementioned Thorpe et al.'s studies, recognition occurred at the superordinate level of specificity. But can the processing of features and feature-conjunctions also lead to such early identification-related activations when objects need to be classified at a more specific entry-level?

Electroencephalography (EEG) provides a measure of fast temporal processes that lead to object recognition and is an ideal tool for assessing the temporal locus of representational processing. In EEG studies, first object-related effects are reported to occur at approx. 80 ms [12], which is in the time range of the first positive component P1 of the event-related potential (ERP). It is considered that early object-related effects in the P1 range are task-independent: a product of low-level perceptual processing of image properties, highly sensitive to changes in luminance, contrast or spatial frequencies [14]. While P1 reflects the earliest evoked component carried by the lower frequency bands of the EEG signal (usually ERPs are filtered at 25 Hz), it partially temporally overlaps with the evoked gamma-band activity (GBA). Evoked GBA is time and phase-locked to stimulus onset; it is evident around 50–150 ms in the lower gamma-band frequency ranges (30–40 Hz). Modulations of its amplitude reflect differences in the perceptual processing of features [15], [16] and it is highly sensitive to changes in low-level properties of images [17], [18]. Using an object/non-object discrimination task, a few studies have obtained object-related modulations of evoked GBA [19], [20], paralleling the early evoked effects seen by the Thorpe group. Induced GBA is non time or phase-locked to stimulus onset. It usually occurs around 200–400 ms and its frequency (30–90 Hz) tends to vary between participants. Significant levels of induced GBA are elicited in studies that require identification of foveally presented familiar objects [21], [22] and it is thought to reflect synchronisation in distributed neural assemblies which is a marker of cortical object representation [23]. A frontal ERP component with a latency of around 200–400 ms known as the N350 is a marker of another late representational process – object model selection, which matches the visual percept to stored object knowledge and is affected by image attributes [24], [25].

**Figure 1:**
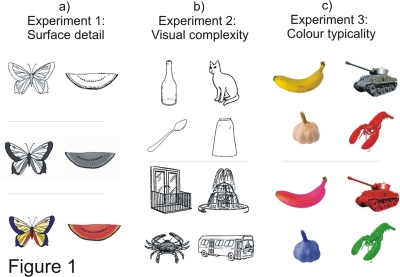
Examples of stimuli. a) Experiment 1: line drawings, gray-shaded and coloured images of objects (taken from Rossion and Pourtois, 2004); b) Experiment 2: low and high visual complexity images of objects (taken from Bates et al., 2003); c) Experiment 3: typically and atypically coloured images of colour-diagnostic objects (taken from Naor-Raz et al., 2003).

To determine the temporal locus of identification-specific modulations in entry-level recognition, we selectively varied the amount and representational role of object features and related them to the electrical activity in the human brain (P1, N350, evoked and induced GBA). In a series of EEG experiments, participants had to successfully identify images of objects that contained different types or different degrees of visual object features (see Figure 1). We examined the representation of surface detail (i.e., shading-defined texture and colour; Experiment 1), visual complexity (i.e., amount of contours; Experiment 2) and colour typicality (i.e., typical or atypical colour of colour diagnostic objects; Experiment 3).

**Figure 2:**
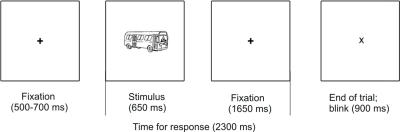
Trial outlook (same for all experiments).

These features play different representational roles: colour facilitates identification (with a particular role for colour diagnostic objects) while additional contours deter it. In the EEG, we focused on modulations of two early markers: the P1 and the evoked GBA. We also assessed the N350 and induced GBA, as markers of late representational activity. Object identification was assessed through a grammatical gender decision task, which required the participants to make a syntactic judgment on the object's name (for trial outlook, see Figure 2). This task was experimentally validated in a previous study [26] and due to it being an implicit naming task, it ensured that object identification was performed at the entry-level of specificity.

## Results

### Experiment 1: Surface detail

#### Design

This experiment relied on the Rossion and Pourtois [1] stimulus set of objects presented at different levels of surface detail, based on the well-known and widely used Snodgrass and Vanderwart [27] set. The set is large, containing objects from many different categories. For each object, three versions of the image are represented: a line, a grey-shaded and a coloured drawing. A line drawing is a specific form of a 2D object-image, because it contains pre-processed edges. Also, the image of a line drawing solely consists of high spatial frequencies. Meanwhile, in addition to those same predefined edges, textured or coloured line drawings also contain information about the surface of the object, given in low spatial frequencies. Therefore, the images in the stimulus set differ both in their low-level visual properties, and in the sources of information that can be used to access the object representation (shape, or shape and colour).

Stimuli consisted of 210 images of familiar objects for each of the three levels of surface detail (line, texture and colour; thus, 630 total images; see Figure 1a); out of these, 67 were colour diagnostic and 143 were non colour diagnostic objects. Each participant was shown a randomly pre-selected subset of stimuli for each of the three conditions – a total of 210 stimuli. By not presenting images of the same object with different level of surface detail to the same participant, previously reported repetition suppression effects in the induced GBA were avoided [21], [28]. Stimulus presentation was balanced across the sample to control for item-specific effects. Thus, across the sample, each object was seen equally often at each level of surface detail (line, texture, colour).

#### Findings

**Figure 3:**
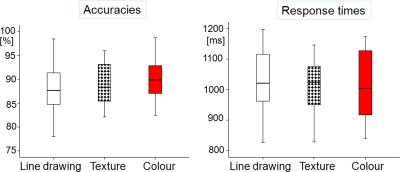
Accuracies and response times for Experiment 1. Data is depicted by box plots, with midlines indicating medians, ends of boxes indicating 25th and 75th percentiles, ends of lines indicating 10th and 90th percentiles, and dots indicating observations falling in the outlying 10 percentiles.

Repeated measures ANOVA revealed no effect on reaction times (F (2, 16) = 0.84, n.s.) or accuracies (F (2, 16) = 0.61, n.s.) in an across participant analysis (see Figure 3). When colour diagnostic objects were analysed in isolation across items, a significant reduction in response times was found (F (2, 65) = 4.28; p<0.05) for coloured images (means: line drawing 1096±18 ms; shaded 1117±20 ms; coloured 1062±18 ms). This demonstrates that colour was indeed processed as a unique attribute of the object image and facilitated object recognition.

**Figure 4 pone-0003781-g004:**
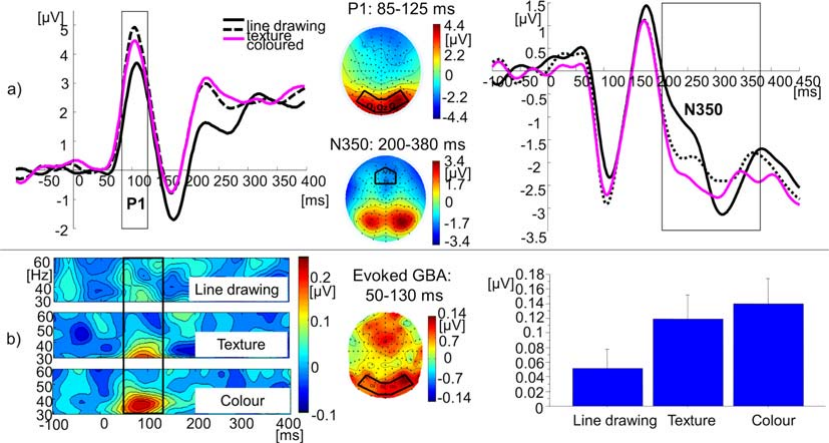
EEG findings from Experiment 1 (surface detail): ERPs and evoked GBA. a) ERPs: Grand mean baseline corrected ERP time courses at regional mean sites with time windows of P1 and N350 components indicated by grey boxes (black: line drawings; dotted: textured; magenta: coloured). To the right, scalp topographies of P1 and N350, averaged across all conditions. b) Evoked GBA: Grand mean baseline-corrected TF-plots averaged across 128 electrodes. Black box indicates the time-window of maximal activity. To the right, grand mean 3D spherical spline amplitude-map representing an average across conditions, based on the ±2.5 Hz frequency band centred on the 35 Hz wavelet during the time-window of maximal activity. Black box indicates the electrode sites of interest. All plots represent the average across the sample. Electrode names are given for channels taken into regional means. Bar plots of grand mean evoked GBA are also given, with a one standard error bar.

In the EEG, we found a highly significant increase of amplitude for both P1 (F (2, 16) = 9.29, p<0.001) and evoked GBA (F (2, 16) = 3.50, p<0.05) when the amount of object features was increased by adding surface detail (see Figure 4). A shift in the latency of N350 was also found (F (2, 16) = 6.06, p<0.05), driven by shorter latencies for coloured pictures as opposed to both line drawings (t (17) = 3.59, p<0.01) and textured drawings (t (17) = 2.21, p<0.05). There was no modulation of N350's amplitude (F (2, 16) = 0.66, n.s.). Induced GBA's amplitude was not modulated (F (2, 16) = 1.63, n.s.).

#### Conclusion

Surface detail increased the amplitude of early evoked activity elicited in our object identification paradigm. A selective latency shift of the N350 for coloured objects was also found, reflecting a facilitatory effect of colour on object recognition. Is the increase in the amplitude of early components also related to this facilitation, or is it an outcome of an increase in the amount of object features to be processed? In the next experiment, we manipulated the amount of visual complexity in the image. The two effects should now dissociate: as high visual complexity has a detrimental effect on recognition, an early mnemonic input would imply that it should evoke less activity than low visual complexity. However, if there is no early mnemonic input so that evoked activity rather reflects low-level feature processing, it should increase with additional contours in high complexity items.

### Experiment 2: Visual Complexity

#### Design

Visual complexity usually denotes the amount of different object-features contained in an image. Here, we define it as the amount of contour-given detail, qualifying the content in the image that needs to be processed in order to recognise the image as that of a familiar object (as per [2]). Visual complexity can be measured either through mean subjective ratings of images' detail, or objectively through jpeg file size. According to Bates et al. [29], it is more accurate to use objective measures of image complexity that are based on digitised file size. This is due to the fact that, unlike subjective visual complexity ratings, objective measures are independent of culture-specific expectations about the “best” way to represent a given concept.

Objects from the International Picture Naming Project (IPNP) stimulus set [29] were selected into groups of low and high visual complexity based on jpeg file size. Stimuli consisted of 172 images of objects: one half was of low and the other half of high visual complexity (for examples, see Figure 1b). IPNP contains 520 pictures with listed German language naming norms for many relevant naming factors. Items were carefully selected into matched and controlled groups of low and high complexity stimuli based on their visual complexity rank. Initially, images showing objects with natural gender (e.g., a rooster or a hen) were removed, as natural gender could selectively facilitate grammatical gender decisions for these items. Subsequently, all items with neutral gender were removed, in order to reduce the number of possible responses to two equally frequent types of response (masculine and feminine). The remaining items were then ranked based on their visual complexity, with 25% of the items at the lower and upper end of the distribution taken into the final stimulus set. Thus, each condition contained 86 images, with 43 objects being of masculine gender and the other 43 of feminine gender. In order to ascertain the differences between conditions in factors relevant for naming, important variables from the IPNP norms were assessed (see Table 1). Across item one-way ANOVA revealed that the two groups did not differ significantly in normative naming times (F (1,170) = 2.41; n.s.) or other important naming-related factors, apart from *visual* complexity. However, differences were found between objects of low and high *conceptual* complexity (i.e., the amount of object-elements necessary to depict the represented concept), with slower naming times for more complex items (F (1, 170) = 6.02, p<0.05).

**Table 1:** Picture-naming norms and average naming RTs across conditions for Experiment 2 (Means±SEs) (** p<0.001).

	Visual Complexity (JPEG file size, kb)	Word Length (in phonological syllables)	Word Complexity (complex or not)	Name agreement (H statistic)	Frequency (Log natural transformation of frequency counts, CELEX)	Average naming RT for German [ms]
Low complexity (n = 86)	7658±161**	2.13±0.09	0.34±0.05	0.67±0.07	2.05±0.13	1090±31
High complexity (n = 86)	27 984±867**	2.27±0.10	0.31±0.05	0.82±0.08	1.90±0.16	1155±29

#### Findings

**Figure 5:**
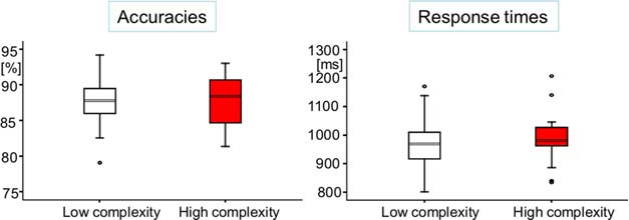
Accuracies and response times for Experiment 2. Data is depicted by box plots, with midlines indicating medians, ends of boxes indicating 25th and 75th percentiles, ends of lines indicating 10th and 90th percentiles, and dots indicating observations falling in the outlying 10 percentiles.

Across participants, we found no differences in response times (t (17) = −1.52, n.s.) or accuracies (t (17) = −0.27, n.s.) between low and high visual complexity stimuli (Figure 5). Significantly higher response times for more complex objects were found in an across item comparison of objects differing in *conceptual* complexity (F (1, 169) = 10.64, p<0.001), confirming that decoding of such images was adversely affected by increased complexity. An across item analysis verified that our response times related to German-language normative naming times by revealing a highly significant correlation (r = 0.74, p<0.001).

**Figure 6:**
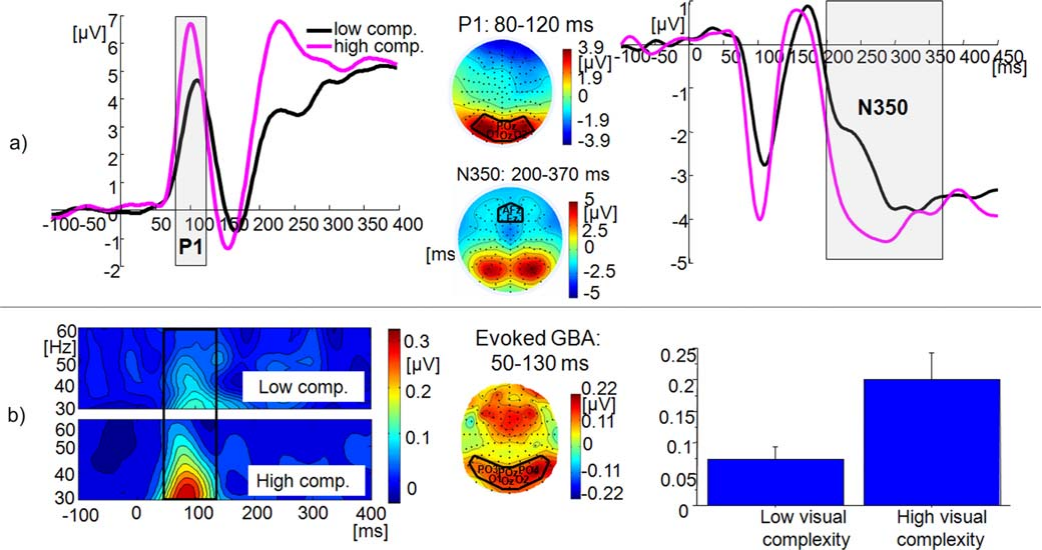
EEG findings from Experiment 2 (visual complexity): ERPs and evoked GBA. a) ERPs: Grand mean baseline corrected ERP time courses at regional mean sites with time windows of P1 and N350 components indicated by grey boxes (black: low visual complexity; magenta: high visual complexity). To the right, scalp topographies of P1 and N350, averaged across conditions. b) Evoked GBA: Grand mean baseline-corrected TF-plots averaged across 128 electrodes. Black box indicates the time-window of maximal activity. To the right, grand mean 3D spherical spline amplitude-map representing an average across conditions, based on the ±2.5 Hz frequency band centred on the 35 Hz wavelet during the time-window of maximal activity. Black box indicates the electrode sites of interest. All plots represent the average across the sample. Electrode names are given for channels taken into regional means. Bar plots of grand mean evoked GBA are also given, with a one standard error bar.

In the EEG it was observed that P1 amplitudes (t (17) = −3.83, p<0.001) and evoked GBA amplitudes (t (17) = −4.19, p<0.001) were increased for visually highly complex items (see Figure 6). Additionally, there was a shift in the latency of the P1 (t (17) = 7.06, p<0.001), with earlier peaks for highly complex objects. N350 amplitude was also more negative for highly complex items (t (17) = 3.88, p<0.001). Induced GBA remained unmodulated (t (17) = −0.10, n.s.).

#### Conclusion

It seems that early evoked activity reflects low-level feature processing since it was again increased with additional object features, despite their detrimental role for object identification. But what would happen if the number of features remained the same and their role for object recognition differed, being either facilitatory or detrimental for the speed of processing? To answer this question, we conducted our final experiment, contrasting identification of colour diagnostic objects which were presented either in their typical colour or in an atypical colour.

### Experiment 3: The typicality of object's colour

#### Design

Colour-shape associations in object recognition are intrinsic: Naor-raz et al. [30] have demonstrated that colour diagnostic objects are recognized faster if presented in their typical colours, while their processing is slowed if the colours are changed to an atypical hue (see Figure 1c). Such a role of visual colour knowledge implies that a yellow banana gives rise to a qualitatively different type of processing than a purple banana – even when the shape in the image is same. In this experiment, we utilized the Naor-raz et al. [30] stimulus set with images of colour diagnostic objects either in their typical colour or in an atypical colour. The stimulus set contained 138 images: each object could be presented either in its typical or in an atypical colour. Typically and atypically coloured objects were counterbalanced across participants, so that each stimulus was shown in each condition an equal number of times across the sample.

#### Findings

**Figure 7:**
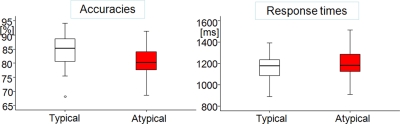
Accuracies and response times for Experiment 3. Data is depicted by box plots, with midlines indicating medians, ends of boxes indicating 25th and 75th percentiles, ends of lines indicating 10th and 90th percentiles, and dots indicating observations falling in the outlying 10 percentiles.

An across participants analysis revealed that RTs were faster for typically coloured objects (typical colour 1150±23 ms, atypical colour 1202±31 ms, t (25) = −2.94, p<0.01). Participants were also more accurate in responses to typically coloured objects (typical colour 84.6±1.2%, atypical colour 80.7±1.0%, t (25) = 3.04, p<0.01; see Figure 7).

**Figure 8:**
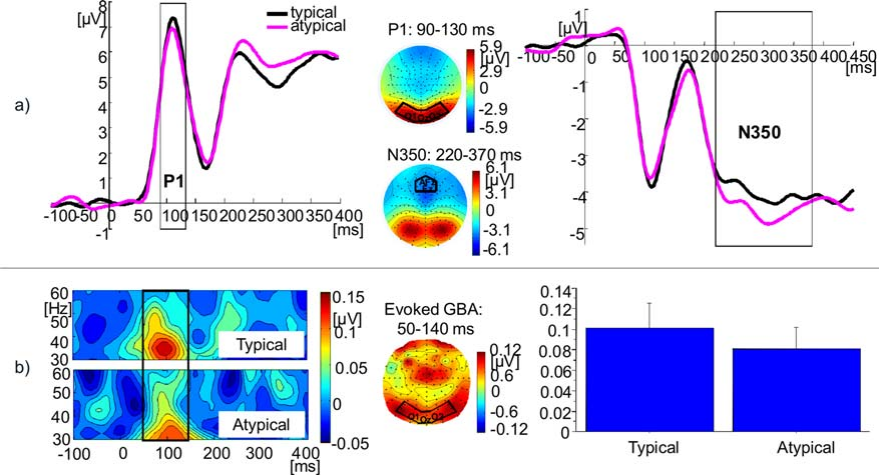
EEG findings from Experiment 3 (colour typicality): ERPs and evoked GBA. a) ERPs: Grand mean baseline corrected ERP time courses at regional mean sites with time windows of P1 and N350 components indicated by grey boxes (black: typical colour; magenta: atypical colour). To the right, scalp topographies of P1 and N350, averaged across conditions. b) Evoked GBA: Grand mean baseline-corrected TF-plots averaged across 128 electrodes. Black box indicates the time-window of maximal activity. To the right, grand mean 3D spherical spline amplitude-map representing an average across conditions, based on the ±2.5 Hz frequency band centred on the 35 Hz wavelet during the time-window of maximal activity. Black box indicates the electrode sites of interest. All plots represent the average across the sample. Electrode names are given for channels taken into regional means. Bar plots of grand mean evoked GBA are also given, with a one standard error bar.

**Figure 9:**
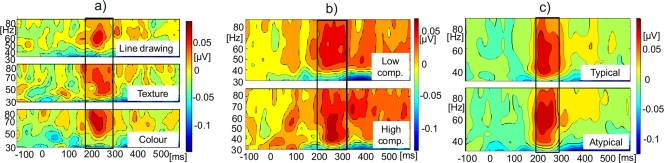
Time-by-Frequency plots for induced GBA (all experiments). a) Experiment 1 - surface detail. b) Experiment 2 - visual complexity; c) Experiment 3 - colour typicality. All plots represent a baseline-corrected grand mean at the sites of maximal activity. Black boxes indicate the time-window of induced GBA response for each condition.

ERP and evoked GBA results are shown in Figure 8. There were no changes in P1 amplitudes (t (25) = 1.28, n.s.) or evoked GBA amplitudes (t (25) = 0.75, n.s.). However, the modulation in N350 amplitude was significant (t (25) = 2.15, p<0.05), with more negativity for atypically coloured objects. Induced GBA remained unmodulated (t (25) = −0.74, n.s.; see Figure 9).

#### Conclusion

In this experiment, with the overall amount of features constant across conditions, the early evoked components remained unmodulated. The shift in N350 again reflected the negative impact of atypical colours on the representational process, in line with the observed behavioural effects. Therefore, we conclude that early evoked components reflect low-level feature processing. Task-relevant mnemonically influenced processing of object features has its earliest effects on the N350 component, a marker of object model selection.

## Discussion

We selectively varied the amount and representational role of object features and related them to the electrical activity of the human brain. The novelty of our study is in the fact that the contribution of object features to representation was ensured by a task that demanded entry-level identification, which is used in everyday perception of real-life objects (trees, houses, dogs, cars, etc.). Therefore, for the first time it was possible to assess if the feature's representational relevance would have a differential impact on the earliest measures of object processing.

The role of features for object representation was confirmed through RT costs or benefits. P1 component of the ERP and evoked GBA's amplitude increased when more image features had to be coded, without reflecting the specific feature's qualitative contribution to the recognition process. The similarity of the effects of surface detail on both the P1 and the evoked GBA indicates that these two components may reflect complementary and co-occurring sensory processing of stimuli. On the contrary, modulations at the level of the ERP component N350 reflected the representational significance of object features. N350 is a component sensitive to image quality and reflects integrative activity that follows the initial processing stream in the visual system. The temporal locus of the feature-specific mnemonic effect was thus at the N350 component, around approx. 200–350 ms. Induced GBA, which co-occurs with the N350, remained unmodulated in its amplitude. Thus, induced GBA reflects stages of processing that do not directly relate to picture decoding processes and may be more conceptual in nature.

Our findings on early evoked effects due to changes in object features extend the findings of the Thorpe group [12] who studied superordinate object identification. They found earliest object-related EEG effects at 80 ms. While these effects were task-independent, there were also task-related but category-independent modulations at approximately 150 ms. Such findings are likely to stem from top-down driven segmentation of the image wherein high-level feature conjunctions are selected on the basis of their diagnosticity for target-category stimuli (vehicles or animals [13]). In our task, participants had to implicitly name images of objects from a large and varied set: there was no segmentation from background involved and specific identity of each object had to be accessed. In such conditions, identification-specific effects cannot emerge as early – they are divergent from both sensory feature-related effects at 80 ms, as well as from category-independent but task-related effects of feature-conjunctions at 150 ms. In our study, identification-specific effects started at approximately 200 ms and were observable at the level of the N350 component of the ERP. Convergent are the findings of Johnson and Olshausen [31] who attributed object-related changes in ERP waveforms prior to 137 ms to early processing of featural differences and late changes post 150 ms to the recognition process itself.

We found that N350 modulations matched the effects obtained in the behavioural data: benefits for coloured objects (earlier latency in Experiment 1), as well as costs for more contours (higher amplitude in Experiment 2) or atypical colour of colour-diagnostic items (higher amplitude in Experiment 3). N350 is known to reflect object-matching processes and responds with higher amplitudes to images that are not as straightforwardly identifiable [24], [25]. N350 and its posterior complement Ncl are thought to be generated in the posterior ventral cortex – in particular, the lateral-occipital complex, a significant part of the ventral recognition stream [32], [33]. Schendan and Kutas [25] propose that N350 reflects a reactivation of occipito-temporal cortex in order to integrate information across a wide range of representational regions, from early visual areas to the ventrolateral prefrontal cortex [34], [35]. Unlike earlier evoked components, it was modulated by the features representational role, indicating that it is the earliest evoked locus of feature-driven mnemonic effects in entry-level object recognition.

Although induced GBA is generally considered to be the earliest marker of cortical object representation, it remained unmodulated in our study. Induced GBA's amplitude has thus been shown to be less sensitive to image attributes that drive perceptual processing of objects. It may be that induced GBA's amplitude is more dependent on the conceptual processing of the object's identity. Such conceptual processing is necessarily intertwined with perceptual processes, as semantic and perceptual levels of representation are known to be coupled [36]. The conceptual nature of induced GBA is supported by the fact that it can also be elicited in word/pseudoword discrimination paradigms with highly similar attributes to peaks elicited with object/non-object tasks. Both with words and visual objects, induced GBA peaks spanned the 200–350 ms period and exhibited repetition suppression effects for familiar words and objects ([21], [22], [37]). The importance of semantic associations for induced GBA elicited by familiar objects is also demonstrated in the fact that even after 10 repetitions, unfamiliar objects (i.e., nonsensical images created by scrambling of familiar objects) do not start exhibiting the sharpening effect associated with repetition suppression [38]. In our experiments, induced GBA was always associated with successful recognition of familiar objects. Thus, no variability in conceptual processes between conditions would be expected and is likely to be reflected in the steady amplitude of the induced GBA across conditions.

An explanation of scalp-recorded induced GBA as a manifestation of miniature or microsaccadic eye movements has recently been put forward [39]. Microsaccades are rapid small-amplitude eye movements spontaneously occurring about once per second with the main purpose of countering perceptual fading, whose role for visual perception and attention is only starting to be explored [40]. Yuval-Greenberg et al. [39] observed that miniature eye movement's saccadic spike potentials and induced GBA are both modulated by object coherence and object type in a highly correlated fashion. In our study, lack of modulation of induced GBA's amplitude by object features in the presence of multiple modulations of other more feature-sensitive components (evoked GBA, ERPs) would indicate that the induced GBA we have observed is different in nature to the ‘miniature saccade’ related activity reported by Yuval-Greenberg et al. [39]. Indeed, an ongoing discussion supports the possibility of accurate scalp-recordings of induced GBA if proper study design (foveal presentation with instruction to suppress eye movements) and artifact removal procedures are used (see comments at Neuron online: http://www.neuron.org/content/article/comments?uidPIIS0896627308003012#top). Thus, we conclude that the communality of object-related conceptual processes between conditions was reflected in the steady amplitude of the induced GBA, irrespective of the changes in lower-level features.

In summary, it is now generally accepted that visual object identification is achieved through a functional cooperation of distributed brain regions, integrating diverse information in a remarkably fast and efficient fashion. In spite of great variability of viewpoints, sizes or possible occlusions in everyday visual scenes, objects are identified within approx. 300 ms of processing time. Models of visual object recognition attempt to explain the rapid and concurrent processes that lead to successful object identification, with an initial feed forward processing stream followed by a series of feedback loops. While the earliest processing, lasting up to 100–150 ms, deals with the analysis of low-level object features and their conjunctions, later stream of processing lasting up to 300 ms is thought to reflect the mnemonic continuation of representational activity [12], [41]. Neurophysiological measures can directly reflect the time course of cognitive processes, making EEG an essential tool in studying differential processing of objects in the human brain during the crucial period of representational processing (i.e., up to 300–400 ms). Our series of EEG experiments systematically explored stimulus space according to object feature's relevance to representational processing at entry-level of specificity, at which object's identity is accessed in everyday life. It demonstrated that in these circumstances, feature-driven mnemonic effects can only appear in a time window that allows recurrent and feedback interactions between representational brain areas [25]. Any earlier effects (i.e. prior to 200 ms) are related to the amount of features in the image and their conjunctions which can be highly diagnostic of object identity under specific tasks and stimulus sets (explaining the findings of [13], [19]). In everyday vision categorical representational processing of objects is not ultra-rapid and seems to require the full 300 ms of neural processing. This has significant implications on models of visual object representation. Recently, pictorial models of object representation which emphasise the differential contribution of image features at several hierarchically organised stages of classification have emerged [42]. The body of EEG findings in object recognition support such feature-based models. Thus, depending on the level of specificity of classification, the contribution of features can be either early -subserving ultra-rapid but coarse categorisation as in the studies of Thorpe and Herrmann research groups- or late -subserving entry-level categorisation that was examined in this study.

## Materials and Methods

### Participants

Healthy university students received class credit or a small honorarium for participating in the study (Experiment 1: 18 participants aged 19–39 years, mean age = 23 years; Experiment 2: 18 participants aged 18–26 years, mean age = 22 years; Experiment 3: 25 participants aged 19–33, mean age 33 years). Participants had been removed from the sample when technical problems had occurred during the recording or if they exhibited excessive EEG artifacts (less than 60% artifact free trials) (Experiment 1: two participants; Experiment 2: three participants; Experiment 3: four participants). Participants reported normal or corrected-to-normal vision and all were native speakers of German. None had participated in object recognition studies in preceding six months. Individual written informed consent was obtained and the study conformed to the Code of Ethics of the World Medical Association.

### Stimulus presentation and task

Stimulus presentation occurred in a random order, which was different for each of the participants. Participants performed an implicit naming task requiring them to press a different button depending on the grammatical gender of object's name (in Experiments 1 and 3 masculine, feminine or neutral; in Experiment 2 masculine or feminine). For a detailed description of the task, see Martinovic et al. [26]. Participants first performed a practice block (in Experiment 1, 32 trials; in Experiment 2, 40 trials; in Experiment 3, 30 trials) – the practice contained a subset of stimuli that were not used in the experiment itself. Experiment 1 consisted of two blocks, each one lasting approximately seven minutes and containing 105 trials. Experiment 2 had four blocks with 43 stimuli, each lasting approx. three minutes. Experiment 3 consisted of two blocks with 69 trials, each lasting approx. four and a half minutes. Each trial consisted of a variable 500–800 ms baseline period, during which a black fixation cross (0.6°×0.6°) was presented. The fixation cross was then removed and a stimulus picture was displayed for 650 ms. The picture was then replaced by the fixation cross, which remained on the screen for another period of 1650 ms. This was followed by the display of an ‘X’ for 900 ms, during which participants were allowed to blink. Stimuli were presented centrally on a 19-inch computer screen, with a 70 Hz refresh rate. The monitor was positioned outside of the dimly lit soundproof testing chamber and the participants viewed it through a window from a 1 m distance. The objects presented on the images subtended a visual angle ranging from around 1.5° to around 4.6°. All stimuli were shown on a white background. Stimulus onset was synchronised to the vertical retrace of the monitor. The presentation and the timing of the experiment were controlled using a Matlab Toolbox, allowing visual presentation and response-recording with precise timing (Cogent, www.vislab.ucl.ac.uk/Cogent/; The Mathworks, Inc, Natick, Massachusetts). Halfway through the experiment participants were asked to change the responding hand. Participants were instructed to minimise eye movements and blinking during the display of a stimulus or the fixation cross.

### EEG recording

EEG was recorded continuously from 128 locations using active Ag-AgCl electrodes (BioSemi Active-Two amplifier system; Biosemi, Amsterdam, The Netherlands) placed in an elastic cap. In this system the typically-used “ground” electrodes in other EEG amplifiers are replaced through the use of two additional active electrodes, positioned in close proximity to the electrode Cz of the international 10–20 system [43]: Common Mode Sense (CMS) acts as a recording reference and Driven Right Leg (DRL) serves as ground [44], [45]. Horizontal and vertical electrooculograms were recorded in order to exclude trials with blinks and significant eye movements. EEG signal was sampled at a rate of 512 Hz and was segmented into epochs starting 500 ms prior and lasting 1500 ms following picture onset. EEG data processing was performed using the EEGlab toolbox [46] combined with in-house procedures running under the Matlab (The Mathworks, Inc, Natick, Massachusetts) environment. Artefact correction was performed by means of “statistical correction of artefacts in dense array studies” (SCADS; [47]). It is widely accepted in the field and has been applied and described in several publications [48], [49]. All incorrectly answered trials were excluded prior to data analysis. In Experiment 1, the average rejection rate was 29.3%, resulting in approx. 44 remaining trials per condition. In Experiment 2, the average rejection rate was 29.6%, resulting in approx. 53 remaining trials per condition. In Experiment 3, the average rejection rate was 26.5%, resulting in approx. 42 remaining trials per condition. Further analyses were performed using the average reference.

### Behavioural data analysis

RTs between 400 and 2300 ms, the maximum time allowed for responses, for trials with correct responses were taken into further analysis. Accuracy rates were analysed across participants while RTs on correctly answered trials were analysed both across participants and across items. Median RTs for correct items were computed for each participant. Means across participants were then computed to obtain a measure of central tendency known as a mean of median RT. This was done due to the skewness of RT distributions and is a common procedure when working with RTs (e.g., see [26]). Across participant differences were analysed with repeated measures ANOVAs or paired t-tests; across item differences were analysed with one-way ANOVAs.

### Event related potentials (ERPs) analysis

A 25 Hz low-pass filter was applied to the data before all ERP analyses. Two ERP components were assessed: P1 and N350. Figures 3–5 (see Results) list the analysis windows and electrode sites taken into the regional mean for each component. Mean amplitude within the respective time window was calculated for each component and mean amplitude during the period 100 ms prior to stimulus onset (baseline) was subtracted. Each component was subject to repeated measures ANOVAs or paired t-tests. Post-hoc tests were performed using paired t-tests.

### Analysis of evoked and induced spectral changes

High frequency oscillatory activity was analysed according to the standard procedure employed in many previous studies (e.g., [21], [22], [26], [50]). In brief, spectral changes in oscillatory activity were analysed by means of Morlet wavelet analysis [51], which offers a good compromise between time and frequency resolution [23]. This method provides a time-varying magnitude of the signal in each frequency band leading to a time-by-frequency (TF) representation of the signal and, together with suggested parameter definitions that allow for a good time and frequency resolution in the gamma frequency range, is detailed in previous studies (e.g. [22]). In order to achieve good time and frequency resolution in the gamma frequency range, the wavelet family in this study was defined by a constant m = f0 /σf = 7, with f0 ranging from 2.5 to 100 Hz in 0.5 Hz steps. This data was subsequently reduced to form 2.5 Hz-wide wavelets. Time-varying energy in a given frequency band was calculated for each epoch by taking the absolute value of the convolution of the signal with the wavelet.

Preliminary electrode sites used for time-by-frequency plots were selected on the basis of previous findings of maximal local gamma power elicited by object identification paradigms; parietal for induced GBA ([21], [22], [52]) and occipital for evoked GBA ([52], [53], [54], [55]). These sites were further readjusted in order to envelop the area of maximal amplitude for data collapsed across conditions in case the observed grand mean topography happened to differ from previous findings.

In order to identify the time window and frequency range of the GBA peaks mean baseline-corrected spectral amplitude (baseline: between 200 and 100 ms prior to stimulus onset) was collapsed together for all conditions and represented in TF-plots in the 30–90 Hz range. The length of the time window of maximal gamma band amplitude was defined based on the observed grand-mean GBA, a common approach in previous studies (e.g., [17], [22]). Maps of oscillatory responses in the ±5 Hz frequency band centred upon the 35 Hz wavelet (for evoked GBA) or maximal activity wavelet for each participant (for induced GBA) during the time window of maximal activity were calculated by means of spherical spline interpolations [56]. Regional means of interest were determined on the basis of grand mean topographies.

Evoked oscillatory activity is by definition time-and phase-locked to stimulus onset and was analysed through a transformation of the unfiltered ERP into the frequency domain. Evoked GBA has low inter-individual variability and in object categorisation studies that use line-drawings it is usually observed at frequencies between 30 and 40 Hz, with maximal activity usually occurring in a narrow time interval around 50–150 ms post stimulus-onset (e.g., [21], [22], [50]). Therefore a ±5 Hz range was taken around a central wavelet of 35 Hz within a time window of 50–150 ms. Due to inter-individual differences in the induced gamma peak in the frequency domain a specific wavelet for each participant was chosen based on the frequency of his/her maximal amplitude in an average across all three conditions. Centred upon this wavelet a frequency band of ±5 Hz was subsequently formed for the purpose of statistical analyses.

Differences between conditions at the regional mean sites in the amplitude after baseline subtraction were analysed by means of repeated measurement ANOVAs or paired t-tests.

## References

[1] Rossion B, Pourtois G (2004). Revisiting Snodgrass and Vanderwart's object pictorial set: The role of surface detail in basic-level object recognition.. Perception.

[2] Ellis AW, Morrison CM (1998). Real age-of-acquisition effects in lexical retrieval.. Journal of Experimental Psychology: Learning, Memory and Cognition.

[3] Marr D, Nishihara HK (1978). Representation and recognition of the spatial organization of three-dimensional shapes.. Proceedings of the Royal Society of London, Series B.

[4] Biederman I (1987). Recognition-by-components: A theory of human image understanding.. Psychological Review.

[5] Tanaka JW, Weiskopf D, Williams P (2001). The role of color in high level vision.. Trends in Cognitive Sciences.

[6] Yip AW, Sinha P (2002). Contribution of color to face recognition.. Perception.

[7] Gegenfurtner KR, Rieger J (2000). Sensory and cognitive contributions of color to the recognition of natural scenes.. Current Biology.

[8] Funnell E, Hughes D, Woodcock J (2006). Age of acquisition for naming and knowing: A new hypothesis.. Quarterly Journal of Experimental Psychology.

[9] Reis A, Faisca L, Ingvar M, Petersson KM (2006). Color makes a difference: Two-dimensional object naming in literate and illiterate subjects.. Brain and Cognition.

[10] Thorpe S, Fize D, Marlot C (1996). Speed of processing in the human visual system.. Nature.

[11] Fize D, Fabre-Thorpe M, Richard G, Doyon B, Thorpe SJ (2005). Rapid categorization of foveal and extrafoveal natural images: Associated ERPs and effects of lateralization.. Brain and Cognition.

[12] VanRullen R, Thorpe SJ (2001). The time course of visual processing: from early perception to decision-making.. Journal of Cognitive Neuroscience.

[13] Rousselet GA, Mace MJM, Thorpe SJ, Fabre-Thorpe M (2007). Limits of event-related potential differences in tracking object processing speed.. Journal of Cognitive Neuroscience.

[14] Regan D (1989). Human brain electrophysiology.

[15] Eckhorn R, Reitboeck HJ, Arndt M, Dicke P (1990). Feature linking via synchronization among distributed assemblies: simulations of results from cat visual cortex.. Neural Computation.

[16] Karakas S, Basar E (1998). Early gamma response is sensory in origin: a conclusion based on cross-comparison of results from multiple experimental paradigms.. International Journal of Psychophysiology.

[17] Busch NA, Debener S, Kranczioch C, Engel AK, Herrmann CS (2004). Size matters: effects of stimulus size, duration and eccentricity on the visual gamma-band response.. Clinical Neurophysiology.

[18] Fründ I, Busch NA, Korner U, Schadow J, Herrmann CS (2007). EEG oscillations in the gamma and alpha range respond differently to spatial frequency.. Vision Reasearch.

[19] Herrmann CS, Lenz D, Junge S, Busch NA, Maess B (2004). Memory-matches evoke human gamma-responses.. BMC Neuroscience.

[20] Fründ I, Busch NA, Schadow J, Gruber T, Körner U (2008). Time pressure modulates electrophysiological correlates of early visual processing.. PLOS Biology.

[21] Gruber T, Malinowski P, Müller MM (2004). Modulation of oscillatory brain activity and evoked potentials in a repetition priming task in the human EEG.. European Journal of Neuroscience.

[22] Gruber T, Müller MM (2005). Oscillatory brain activity dissociates between associative stimulus content in a repetition priming task in the human EEG.. Cerebral Cortex.

[23] Tallon-Baudry C, Bertrand O (1999). Oscillatory gamma activity in humans and its role in object representation.. TICS.

[24] Schendan HE, Kutas M (2003). Time course of processes and representations supporting visual object identification and memory.. Journal of Cognitive Neuroscience.

[25] Schendan HE, Kutas M (2007). Neurophysiological evidence for the time course of activation of global shape, part, and local contour representations during visual object categorization and memory.. Journal of Cognitive Neuroscience.

[26] Martinovic J, Gruber T, Hantsch A, Müller MM (2008). Induced gamma-band activity is related to the time point of object identification.. Brain Research.

[27] Snodgrass JG, Vanderwart M (1980). A standardized Set of 260 pictures: Norms for name agreement, image agreement, familiarity and visual complexity.. Journal of Experimental Psychology: Human Learning and Memory.

[28] Gruber T, Müller MM (2002). Effects of picture repetition on induced gamma band responses, evoked potentials, and phase synchrony in the human EEG.. Cognitive Brain Research.

[29] Bates E, D'Amico S, Jacobsen T, Szekely A, Andonova E (2003). Timed picture naming in seven languages.. Psychonomic Bulletin & Review.

[30] Naor-Raz G, Tarr MJ, Kersten D (2003). Is color an intrinsic property of object representation?. Perception.

[31] Johnson JS, Olshausen BA (2003). Timecourse of neural signatures of object recognition.. Journal of Vision.

[32] Schendan HE, Kutas M (2002). Neuropsychological evidence for two processing times for visual object identification.. Neuropsychologia.

[33] Doniger GM, Foxe JJ, Murray MM, Higgins BA, Snodgrass JG (2000). Activation timecourse of ventral visual stream object-recognition areas: High density electrical mapping of perceptual closure processes.. Journal of Cognitive Neuroscience.

[34] David O, Harrison L, Friston KJ (2005). Modelling event-related responses in the brain.. Neuroimage.

[35] Brincat SL, Connor CE (2006). Dynamic shape synthesis in posterior inferotemporal cortex.. Neuron.

[36] van Schie HT, Wijers AA, Kellenbach ML, Stowe LA (2003). An event-related potential investigation of the relationship between semantic and perceptual levels of representation.. Brain and Language.

[37] Fiebach CJ, Gruber T, Supp GG (2005). Neuronal mechanisms of repetition priming in occipitotemporal cortex: spatiotemporal evidence from functional magnetic resonance imaging and electroencephalography.. Journal of Neuroscience.

[38] Conrad N, Giabbiconi CM, Müller MM, Gruber T (2007). Neuronal correlates of repetition priming of frequently presented objects: Insights from induced gamma band responses.. Neuroscience Letters.

[39] Yuval-Greenberg S, Tomer O, Keren AS, Nelken I, Deouell LY (2008). Transient induced gamma-band response in EEG as a manifestation of miniature saccades.. Neuron.

[40] Engbert R, Martinez-Conde S, Macknik S, Martinez L, Alonso J-M, Tse P (2006). Microsaccades: A microcosm for research on oculomotor control, attention and visual perception.. Fundamentals of vision: Low and mid-level processes in perception.

[41] Bar M, Kassam KS, Ghuman AS, Boshyan J, Schmid AM (2006). Top-down facilitation of visual recognition.. Proceedings of the National Academy of Sciences of USA.

[42] Ullman S (2007). Object recognition and segmentation by a fragment-based hierarchy.. Trends in Cognitive Sciences.

[43] Jasper HH (1958). The 10–20 electrode system of the International Federation.. Electroencephalography and Clinical Neurophysiology.

[44] Metting Van Rijn AC, Peper A, Grimbergen CA (1990). High quality recording of bioelectric events: I: interference reduction, theory and practice.. Medical and Biological Engineering and Computing.

[45] Metting Van Rijn AC, Peper A, Grimbergen CA (1991). High quality recording of bioelectric events. II: a low noise low-power multichannel amplifier design.. Medical and Biological Engineering and Computing.

[46] Delorme A, Makeig S (2004). EEGLAB: an open source toolbox for analysis of single-trial EEG dynamics including independent component analysis.. Journal of Neuroscience Methods.

[47] Junghoefer M, Elbert T, Tucker DM, Braun C (2000). Statistical control of artifacts in dense array EEG/MEG studies.. Psychophysiology.

[48] Gruber T, Müller MM, Keil A, Elbert T (1999). Selective visual-spatial attention alters induced gamma band responses in the human EEG.. Clinical Neurophysiology.

[49] Muller MM, Keil A (2004). Neuronal synchronization and selective color processing in the human brain.. Journal of Cognitive Neuroscience.

[50] Martinovic J, Gruber T, Müller MM (2007). Induced gamma-band responses predict recognition delays during object identification.. Journal of Cognitive Neuroscience.

[51] Bertrand O, Pantev C, Pantev C, Elbert T, Lütkenhöner B (1994). Stimulus frequency dependence of the transient oscillatory auditory evoked response (40 Hz) studied by electric and magnetic recordings in human.. Oscillatory Event-Related Brain Dynamics.

[52] Martinovic J, Gruber T, Müller MM (2007). Induced gamma-band responses predict recognition delays during object identification.. Journal of Cognitive Neuroscience.

[53] Tallon-Baudry C, Bertrand O, Delpuech C, Pernier J (1997). Oscillatory gamma-band (30–70 Hz) activity induced by a visual search task in human.. The Journal of Neuroscience.

[54] Busch NA, Herrmann CS, Müller MM, Lenz D, Gruber T (2006). A cross-lab study of event-related gamma activity in a standard object-recognition paradigm.. Neuroimage.

[55] Herrmann CS, Mecklinger A, Pfeifer E (1999). Gamma responses and ERPs in a visual classification task.. Clinical Neurophysiology.

[56] Perrin F, Pernier J, Bertrand O, Echallier JF (1988). Spherical splines for scalp potential and current source density mapping.. Electroencephalography and Clinical Neurophysiology.

